# Delivering on Antimicrobial Resistance Agenda Not Possible without Improving Fungal Diagnostic Capabilities

**DOI:** 10.3201/eid2302.152042

**Published:** 2017-02

**Authors:** David W. Denning, David S. Perlin, Eavan G. Muldoon, Arnaldo Lopes Colombo, Arunaloke Chakrabarti, Malcolm D. Richardson, Tania C. Sorrell

**Affiliations:** Global Action Fund for Fungal Infections, Geneva, Switzerland (D.W. Denning, D.S. Perlin, A.L. Colombo, A. Chakrabarti, M.D. Richardson, T.C. Sorrell);; he University of Manchester, Manchester, UK (D.W. Denning, E.G. Muldoon, M.D. Richardson);; University Hospital of South Manchester, Manchester (D.W. Denning, E.G. Muldoon, M.D. Richardson);; Rutgers Biomedical and Health Sciences, Newark, New Jersey, USA (D.S. Perlin);; Universidade Federal de São Paulo, São Paulo, Brazil (A.L. Colombo);; Postgraduate Institute of Medical Education and Research, Chandigarh, India (A. Chakrabarti);; University of Sydney, Sydney, Australia (T.C. Sorrell)

**Keywords:** Aspergillus, Candida, Pneumocystis, Histoplasma, Cryptococcus, antimicrobial resistance, antimicrobial drugs, antibiotic drugs, antifungal drugs, fungi, bacteria, HIV/AIDS and other retroviruses, tuberculosis and other mycobacteria, respiratory infections

## Abstract

Antimicrobial resistance, a major public health concern, largely arises from excess use of antibiotic and antifungal drugs. Lack of routine diagnostic testing for fungal diseases exacerbates the problem of antimicrobial drug empiricism, both antibiotic and antifungal. In support of this contention, we cite 4 common clinical situations that illustrate this problem: 1) inaccurate diagnosis of fungal sepsis in hospitals and intensive care units, resulting in inappropriate use of broad-spectrum antibacterial drugs in patients with invasive candidiasis; 2) failure to diagnose chronic pulmonary aspergillosis in patients with smear-negative pulmonary tuberculosis; 3) misdiagnosis of fungal asthma, resulting in unnecessary treatment with antibacterial drugs instead of antifungal drugs and missed diagnoses of life-threatening invasive aspergillosis in patients with chronic obstructive pulmonary disease; and 4) overtreatment and undertreatment of *Pneumocystis* pneumonia in HIV-positive patients. All communities should have access to nonculture fungal diagnostics, which can substantially benefit clinical outcome, antimicrobial stewardship, and control of antimicrobial resistance.

Antimicrobial resistance (AMR) is a major public health concern and a major threat to modern medicine (*1*). In the United States, it is estimated that antibiotic-resistant infections are associated with 23,000 deaths per year (*2*) and excess healthcare-associated costs of approximately US $20–25 billion (*3*). Minimizing AMR has been the focus of accelerating efforts with multipronged approaches tailored to individual countries and healthcare settings. Even if the difficult task of developing new antimicrobial drugs is successful, current efforts aimed at reducing the development of resistance will need to be maintained to protect these novel compounds.

A central tenet of controlling AMR is antibiotic drug stewardship, which seeks to limit inappropriate antibiotic drug usage by avoiding unnecessary prescribing, including discontinuing antibiotic therapy if it is not required. Within the context of stewardship programs, inadequate attention has been paid to fungal infection as the cause of antibacterial treatment failure. Furthermore, the importance of the accurate and timely diagnosis of fungal infections in defeating AMR has been starkly absent from policy discussions (*4*). Accurate diagnosis or exclusion of fungal infection will have a substantial effect on antimicrobial drug usage and on our ability to limit AMR to bacteria.

Few infections are microbiologically diagnosed in real time, so, in general, physicians empirically prescribe most antimicrobial drugs. Furthermore, most infections are never microbiologically confirmed, which matters little if the patient improves and infection resolves, but if the patient’s condition deteriorates, additional empiric antimicrobial drugs are usually given (*5*). Fungal infections, a frequent fatal complication (superinfection) of numerous diseases that contribute to inappropriate antibiotic drug usage (e.g., cancer; liver, respiratory, and renal failure; sepsis; AIDS), are often undiagnosed and untreated.

This inappropriate use of antibiotic drugs must stop. We provide 4 examples of specific clinical situations that require greater application of existing fungal diagnostics and improved overall fungal diagnostic capability and are in line with the 95–95 by 2025 Roadmap from the Global Action Fund for Fungal Infections (*6*).

## Accurate Diagnosis of Fungal Sepsis in Hospitals

Hospitalized patients, especially those in intensive care units (ICUs), are often inappropriately placed on broad-spectrum antibiotic drugs because fungal diseases involving *Candida* spp. are not routinely diagnosed. Bloodstream infection and invasive candidiasis are substantially more common than realized and probably result from multiple factors, including unrestrained antibiotic drug use, indwelling devices, increasing populations of immunocompromised patients, and increased renal support. Multiple studies have shown the incidence of bloodstream infections with *Candida* spp. to be 1.2–26 cases/100,000 population, and the highest rates are in middle- and high-income countries, notably the United States (*7*,*8*). Healthcare-associated infections account for 93% of these infections (≈80% are among hospital inpatients), and the other 7% are community-acquired (*9*). In Brazil, which has a population of 194 million persons, *Candida* bloodstream infections are seen in 14.9 persons/100,000 population, which translates to 29,000 infected persons each year (*10*), based on prospective data obtained 10 years ago from 11 medical centers (*11*). A 2015 prospective study from 27 ICUs in India showed a mean incidence of 6.51 cases of ICU-acquired candidemia per 1,000 ICU admissions and a death rate of 35%–75% (*12*). An estimated 14.3 million patients are admitted to ICUs in India each year. Undoubtedly, this high rate of ICU-acquired candidemia is an underestimate of the problem because blood culture is only ≈40% sensitive for invasive candidiasis (including intraabdominal candidiasis), and use of fluconazole and echinocandins substantially reduces the yield from blood culture (*13*–*15*). Therefore, it is probable that the actual number of cases in ICUs in India exceeds 200,000, resulting in ≈100,000 deaths. If, as is found in other countries, bloodstream infection caused by *Candida* spp. (and presumably invasive candidiasis) outside ICUs in India are at least twice as common as in ICUs, then >600,000 persons each year in India are estimated to have invasive candidiasis. Assuming these patients are treated, ≈300,000 die each year, but many more die if patients are not treated.

In addition, a high prevalence of candidemia has been reported in children, including neonates, in India and Latin America. Central nervous system involvement is common in premature infants, leading to a high rate of neurologic sequelae (*16*).

Although invasive candidiasis is strongly associated with prior bacterial infection and antibacterial therapy, inappropriate escalation and combination antibacterial therapy will typically have been administered to patients with invasive candidiasis. In a study of 444 patients with *Candida* spp. bloodstream infections, 81% were exposed to multiple antibacterial drugs, either concomitantly or sequentially (*17*), and in an ICU study from India, 95% of patients were receiving antibiotic drugs (usually >2) (*12*). Early therapy of *Candida* spp. bloodstream infection greatly improves patient outcomes and the outcome is even better if correct therapy is given immediately (*18*).

Once *Candida*-associated sepsis is confirmed, antibacterial agents can usually be stopped and, if *Candida* sepsis is ruled out, empiric antifungal therapy can be stopped. Inflammation without infection requires no antimicrobial therapy. Three well-validated diagnostic tools, 2 of which are configured for ruling out a diagnosis of invasive candidiasis, are now available: the 1,3 β-D-glucan assay (*19*); the *Candida albicans* germ tube antibody test, which is used with serum samples (*20*); and a nonculture-based molecular assay (newly approved by the Food and Drug Administration) that is used with EDTA blood and is substantially more sensitive than blood culture for making a diagnosis of *Candida* spp. infection (*21*). Among patients without invasive candidiasis, antimicrobial stewardship programs based on such diagnostics have successfully curtailed the use of antifungal therapy in the ICU without worsening patient outcomes (*18*,*22*). The economics of these methods depend on the cost of diagnostic reagents and testing, antifungal drug costs, and incidence of infection (*23*). Overuse of antifungal agents is costly, can promote antifungal resistance, and has the potential for causing toxicity and various detrimental drug interactions in patients (*18*). Modeling will be required to determine the magnitude of the effect of these diagnostic tools on antibacterial prescribing. What is not in dispute is that outcomes for patients with fungal infection will improve. Antimicrobial stewardship programs will be even more effective if these diagnostic tools, with their rapid turnaround times, are readily available (*24*). Widespread implementation of rapid nonculture diagnostics for *Candida* spp. will greatly improve prescribing practices for hospitalized patients with multiple concurrent conditions and poorly functioning organs and using multiple medications.

## Misdiagnosis of Smear-Negative Pulmonary Tuberculosis as Tuberculosis

Smear-negative pulmonary tuberculosis (TB) is a problematic area for clinicians and policymakers. Post-TB sequelae are common, are poorly studied, and may be mistaken for active, recurrent TB (*25*). An apparent underrecognized issue for patients with smear-negative TB is chronic pulmonary aspergillosis (CPA), which can mimic the signs and symptoms of TB. In 544 patients in the United Kingdom who had previously received treatment for TB with a residual cavity, precipitating antibodies to *Aspergillus fumigatus* developed in 24.6% at 2 years and in 34.0% at 5 years. Within 2 years, aspergilloma, a late stage of CPA, developed in 78 (58%) of the 134 patients with precipitating antibody to *A. fumigatus* (*26*). Few prospective studies have been conducted on CPA after treatment for TB, so the incidence of such cases cannot be stated with certainty; conservatively, however, a rate of ≈10% among survivors of pulmonary TB is likely and a global prevalence of ≈1.2 million cases is probable (*26*).

Culture for *Mycobacterium tuberculosis* in samples from smear-negative patients is slow, and results may be falsely negative. The use of new, highly sensitive, DNA detection assays (e.g., Xpert MTB/RIF) directly on respiratory specimens has transformed the rapidity of detecting positive samples, but there remain millions of unwell, smear-negative, PCR-negative patients. Some of these patients have relapsed after anti-TB therapy, and CPA has developed subsequent to cured TB. Among HIV-positive persons, those with smear-negative TB test results have a higher death rate than those with smear-positive results (*27*), probably because many do not have TB at all. It is increasingly recognized that many of these patients are chronically infected with *Aspergillus* spp., resulting in CPA that is largely undiagnosed and untreated.

**Figure 1:**
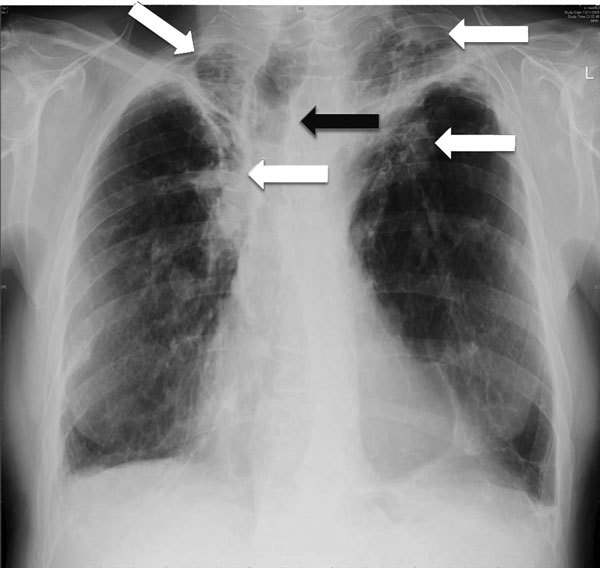
Chest radiograph showing bilateral upper lobe chronic pulmonary aspergillosis, which can be easily mistaken for pulmonary tuberculosis. White arrows indicate areas of abnormality (some pleural thickening and opacification) in both apices, which are similar, although slightly more obvious, to findings in pulmonary tuberculosis. Black arrow indicates the trachea pulled to one side by the contraction and fibrosis on that side. Image used with permission of David Denning (©2016, all rights reserved).

**Figure 2:**
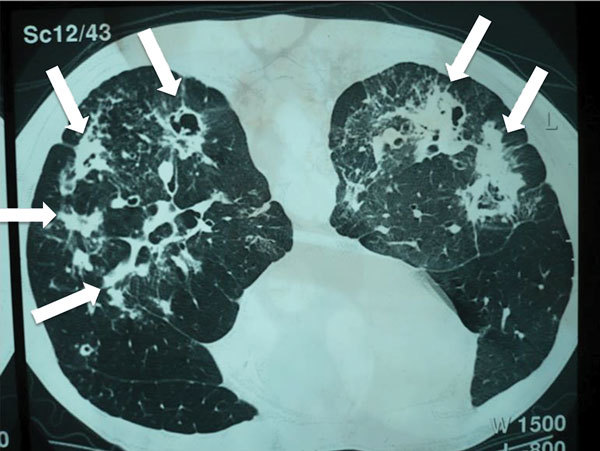
Computed tomography radiograph of thorax showing chronic pulmonary histoplasmosis with bilateral cavitary infiltrates resembling pulmonary tuberculosis, coccidioidomycosis, paracoccidioidomycosis, and aspergillosis. Arrows indicate areas of abnormality. Image used with permission of Arnaldo Colombo (©2016, all rights reserved).

Weight loss, worsening cough, chest pains, dyspnea, and fatigue are common manifestations of TB and CPA, and abnormalities seen on chest radiographs are similar for the 2 diseases (Figure 1). In studies from the United Kingdom, Brazil, South Korea, Iran, and India, the frequency of elevated serum levels of *Aspergillus* antibody after TB varied upwards from 20% (*28*,*29*). *Aspergillus* antibody detection is the key diagnostic test for CPA; the test has 96%–97% sensitivity and 92%–98% specificity (*30*,*31*). Given that CPA is a common sequela to TB and has a 5-year death rate of 75%–80%, it needs to be sought actively by *Aspergillus* antibody testing in symptomatic patients who have completed antituberculous therapy (*32*). A study from Iran showed that serum samples from almost all patients thought to have recurrent TB were positive for *Aspergillus* antibody (*33*). In Brazil and Uganda, *Pneumocystis jirovecii* DNA was identified in the sputum of up to 7.0% and 6.8% of patients, respectively, diagnosed with smear-negative TB. This finding and the recognition of patients with pulmonary histoplasmosis (Figure 2) or coccidioidomycosis (*5*) indicate that other, potentially treatable diseases, not smear-negative TB, may be responsible for illness attributed to TB.

Empiric anti-TB therapy is unnecessary in patients with CPA or other fungal infections because it is ineffective and exposes the patient to potential toxicity. Yet, this therapy remains the default approach for most patients with clinical features and radiologic findings partially consistent with TB. When such treatment fails, as it usually does, there is a risk that patients are assumed to have multidrug-resistant TB, and the inappropriate substitution of second- and third-line anti-TB agents adds to potential toxicities and healthcare costs. The size of this problem is substantial; the World Health Organization reported a total of 2,755,870 patients with smear-negative TB in 2013 (*34*). *Aspergillus* antibody testing should be made widely available and integrated into TB control programs, and emphasis should be placed on the possibility of CPA as a post-TB sequela. All patients with symptoms consistent with recurrent TB should be screened for antibody to *Aspergillus* spp.

## Fungal Exacerbation of Asthma and Chronic Obstructive Pulmonary Disease

It is common practice to treat asthma and exacerbations of chronic obstructive pulmonary disease (COPD) with antibiotic drugs and corticosteroids, although guidelines caution against their unnecessary use, especially in patients with COPD (*35*). Most patients respond, even if the exacerbation is virus-induced. Patients with moderate and severe COPD are frequently hospitalized and have an in-hospital death rate of 6%. More than 80% of patients are treated with antibiotic drugs (*36*), although multiple guidelines advise against this in the absence of purulent sputum and pulmonary infiltrates. It is now well established that colonization (or infection) of the airways with *Aspergillus* spp. is strongly associated with exacerbations (*37*). A study in Spain found that 1.3% of hospitalized patients with COPD had invasive aspergillosis, and 65% died (*38*); in southern China, the frequency rate was 3.9%, and 43% died (*39*). These rates are probably underestimates because they were based on the results of *Aspergillus* sputum cultures, which are insensitive. Most patients do not receive treatment for invasive aspergillosis, but they are treated unnecessarily with antibiotic drugs. The scale of this problem is large. In China, an estimated 11,858,000 COPD patients >40 years of age (87 persons/10,000 population) were hospitalized in 2012 (*40*,*41*); invasive aspergillosis may have developed in 3.9% of those patients (≈462,000 patients of ≈11,858,000 total), and 43% of them (≈200,000 patients of ≈462,000 total) may have died. Presumably other countries with high COPD rates, such as Hungary, Ireland, and New Zealand, face a similar situation.

Persons with asthma who have frequent exacerbations, poorly controlled disease, or both may take several antibiotic drug courses a year, and disease in some of these patients is managed with long-term antibiotic drug therapy. The term fungal asthma has recently been introduced as a catch-all for asthma exacerbated by fungal sensitization, airways fungal colonization in asthma, and/or allergic bronchopulmonary aspergillosis complicating asthma. Awareness and understanding of fungal asthma has recently increased, and the timely diagnosis of these cases and the use of antifungal therapy could substantially reduce the inappropriate use of antibiotic drugs. Long-term antifungal therapy is efficacious in 60%–80% of asthma patients with fungal exacerbations, most notably allergic bronchopulmonary aspergillosis and severe asthma with fungal sensitization (*42*). Successful antifungal therapy results in a reduction in the use of antibiotic drugs and corticosteroids and may reduce hospitalizations.

Diagnosis of fungal asthma relies on total and fungal-specific IgE testing or skin-prick testing, which is simple to perform but not often used. Rapid antigen detection with a lateral flow device might accelerate diagnosis, but its utility for sputum testing or for patients with COPD is unknown. Few studies have addressed the role of fungi in precipitating exacerbations of COPD, but culture and antigen and *Aspergillus* spp. IgG testing all contribute to the diagnosis of chronic and invasive aspergillosis.

Failure to properly diagnose and treat patients with asthma and COPD who are colonized with *Aspergillus* spp. continues to increase the inappropriate use of antibiotic drugs and corticosteroids among these patients. Recognition of fungal infection and allergy and treatment with directed antifungal therapy would greatly reduce exacerbations, medical consultations, and hospital admissions. It is critical that fungal culture and nonculture diagnostics (i.e., *A. fumigatus* IgE, IgG, and antigen testing and PCR) for COPD and asthma exacerbations be evaluated and implemented and that fungal asthma be properly diagnosed and treated.

## Making and Excluding the Diagnosis of *Pneumocystis* Pneumonia in AIDS

**Figure 3:**
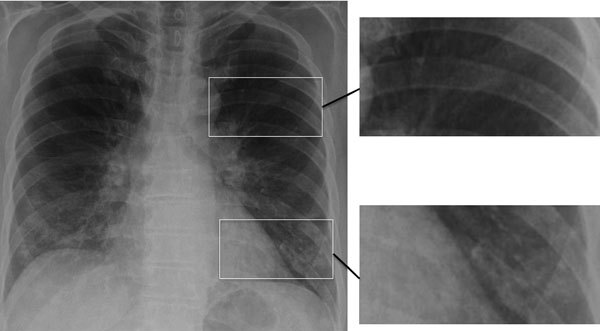
Chest radiograph showing early, subtle *Pneumocystis* pneumonia–associated abnormalities in both lower lungs of a patient newly diagnosed with AIDS; this diagnosis was unsuspected in the patient, a 63-year-old married man. Magnified images on right show normal lung (top image) and infiltrates adjacent to and behind the heart and overlain by rib (bottom image). Similar differences between the upper and lower lobes are seen in the radiograph on the left. Image used with permission of David Denning (©2016, all rights reserved).

*Pneumocystis* pneumonia (PCP) in AIDS is often diagnosed empirically based on a subacute onset of cough; breathlessness out of proportion to abnormalities seen on chest radiographs; and subtle, bilateral changes seen on chest radiographs, in the context of a low CD4 cell count (Figure 3). Co-trimoxazole (trimethoprim/sulfamethoxazole, Bactrim, Septrin) is the most effective agent for prevention and therapy of PCP. A low dose is effective for prophylaxis, but a 3-week course of high and potentially toxic doses is required for effective therapy. The differential diagnosis of PCP is broader in children because bacterial pneumonia is more common among them. If a precise diagnosis could be achieved in most cases of PCP, much of the inappropriate use of co-trimoxazole could be prevented.

Rates of PCP among newly hospitalized adults with advanced HIV infection are highly variable, ranging from <1% to 60%, and rates rise as gross domestic product increases (*43*). Without the availability of adequate diagnostics, many persons will unnecessarily receive high-dose co-trimoxazole, with or without corticosteroids, for 3 weeks. If the actual number of PCP cases in patients with AIDS is 400,000, then hundreds of thousands of hospitalized HIV patients may be given co-trimoxazole unnecessarily, and toxicity rates among them could be as high as 90% (*5*,*44*). Early detection and diagnosis of PCP can help prevent unnecessary hospitalizations and reduce adverse events and healthcare costs. 

Currently, bronchoscopy and microscope examination of bronchoalveolar lavage fluid is the most common definitive means of establishing a diagnosis of PCP; this method has a sensitivity of 75%–90%, depending on the microscopy technique (*45*). *P. jirovecii* fungus is nonculturable in routine laboratories; in Europe, it is commonly molecularly detected using PCR, which has a sensitivity of 95%–99% (*46*). *Pneumocystis* PCR performed on expectorated sputum is also effective for detecting *P. jirovecii* fungus (*47*–*49*), but this method is infrequently used. For children who are breathless, PCR of nasopharyngeal aspirates is currently the only realistic means of establishing a diagnosis. 1,3 β-D-glucan is detectable in the serum of nearly all patients with PCP (*19*); if a sample is negative, infection is effectively ruled out.

Assuming that 25% of PCP cases are mild, immediate diagnosis and use of oral therapy will potentially avoid 100,000 hospital admissions each year and even more if the diagnosis is ruled out and patients are not admitted for unnecessary PCP therapy (*5*). Mild PCP responds well to treatment and prevents progression to moderate or severe infection.

Provision of rapid diagnostics for *Pneumocystis* pneumonia will enable early diagnosis and discontinuation of broad-spectrum antibiotic drugs if test results are positive and discontinuation of high-dose co-trimoxazole and corticosteroids if results are negative. Furthermore, PCP diagnoses that are missed because of concurrent bacterial infection will be minimized. *Pneumocystis* PCR should be used to test all respiratory samples in laboratories serving large immunocompromised populations, and 1,3 β-D-glucan testing should be used on serum in high-volume laboratories. These diagnostics will definitively improve the outcome for immunocompromised patients without AIDS for the same reasons they will improve the outcome for patients with AIDS.

## Other Clinical Scenarios

**Figure 4:**
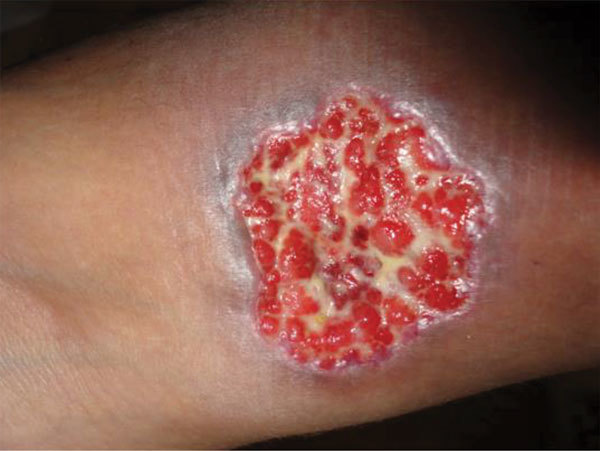
An ulcerative skin lesion that was positive for *Cryptococcus neoformans* fungus on biopsy. For several weeks before being correctly diagnosed, the lesion was misdiagnosed as a bacterial infection. Image used with permission of Arnaldo Colombo (©2016, all rights reserved).

We have not addressed multiple other clinical situations in which a precise fungal diagnosis could reduce the inappropriate prescribing of antimicrobial drugs (i.e., overtreatment or incorrect treatment). Among those situations are cases of cryptococcal meningitis (Figure 4); *Candida* infection or colonization of the respiratory or urinary tract; febrile neutropenia in leukemia; *Aspergillus* bronchitis in bronchiectasis; and allergic, chronic, and invasive fungal sinusitis and PCP in HIV-negative patients. In all these clinical situations, inappropriate antibacterial or antifungal prescribing is common because of the lack of adequate fungal diagnostic testing. We also have not addressed rapid detection of antifungal drug–resistant fungi (i.e., *A. terreus*, *C. krusei*) or the use of diagnostics to prevent superinfection with fungi that are commonly resistant to antifungal drugs, such as *C. glabrata* or *Rhizopus oryzae*. Antifungal drug resistance is problematic in some settings and demands informed prescribing, therapeutic drug monitoring, and development of new antifungal agents (*50*).

## Conclusions

The lack of availability and underuse of nonculture fungal diagnostics results in overprescribing, prescription of unduly long courses of antibacterial agents, and excess empirical use of antifungal agents and leaves many millions of patients with undiagnosed fungal infections. This lack and underuse of proper diagnostics squanders resources. The large scale of the problem, even in many of the world’s most advanced medical centers, compromises AMR control. In many countries, the government and private healthcare providers should be actively promoting diagnosis of fungal infections to minimize deaths and illness from fungal disease; such efforts will probably also have a positive benefit on inappropriate antibacterial drug usage and support stewardship programs. Public health authorities must embrace acute and chronic fungal disease as areas of considerable need and seize the opportunity to improve health and preserve what remains of the antimicrobial drug toolbox.
